# Automated Sensing System for Real-Time Recognition of Trucks in River Dredging Areas Using Computer Vision and Convolutional Deep Learning

**DOI:** 10.3390/s21020555

**Published:** 2021-01-14

**Authors:** Jui-Sheng Chou, Chia-Hsuan Liu

**Affiliations:** Department of Civil and Construction Engineering, National Taiwan University of Science and Technology, Taipei 106, Taiwan; M10705102@mail.ntust.edu.tw

**Keywords:** automated sensing system, dredging construction site, computer vision, convolutional neural networks, object detection, multi-class classification, image recognition

## Abstract

Sand theft or illegal mining in river dredging areas has been a problem in recent decades. For this reason, increasing the use of artificial intelligence in dredging areas, building automated monitoring systems, and reducing human involvement can effectively deter crime and lighten the workload of security guards. In this investigation, a smart dredging construction site system was developed using automated techniques that were arranged to be suitable to various areas. The aim in the initial period of the smart dredging construction was to automate the audit work at the control point, which manages trucks in river dredging areas. Images of dump trucks entering the control point were captured using monitoring equipment in the construction area. The obtained images and the deep learning technique, YOLOv3, were used to detect the positions of the vehicle license plates. Framed images of the vehicle license plates were captured and were used as input in an image classification model, C-CNN-L3, to identify the number of characters on the license plate. Based on the classification results, the images of the vehicle license plates were transmitted to a text recognition model, R-CNN-L3, that corresponded to the characters of the license plate. Finally, the models of each stage were integrated into a real-time truck license plate recognition (TLPR) system; the single character recognition rate was 97.59%, the overall recognition rate was 93.73%, and the speed was 0.3271 s/image. The TLPR system reduces the labor force and time spent to identify the license plates, effectively reducing the probability of crime and increasing the transparency, automation, and efficiency of the frontline personnel’s work. The TLPR is the first step toward an automated operation to manage trucks at the control point. The subsequent and ongoing development of system functions can advance dredging operations toward the goal of being a smart construction site. By intending to facilitate an intelligent and highly efficient management system of dredging-related departments by providing a vehicle LPR system, this paper forms a contribution to the current body of knowledge in the sense that it presents an objective approach for the TLPR system.

## 1. Introduction

Dredging operations are an essential part of hydraulic engineering in Taiwan. In such work, the extraction and sale of sand and gravel involve considerable profits. Therefore, illegal activities like sand and gravel theft, violence, and intimidation are not uncommon [1,2]. These incidents seriously affect homeland security, leading to the limited effectiveness of flood control for which a budget is planned annually by the government. Hence, the monitoring and management of dredging operations have become critical tasks that are continually monitored by the relevant government’s departments and agencies.

Generally, hundreds of dump trucks enter and exit a construction area daily, and personnel must accurately control them such that inspections do not affect construction progress. Although patrol officers and security guard personnel in construction areas manually perform frontline control at control points at the exits and entrances thereof [3], inspections and checks may be lax owing to the temptation of bribes and violent threats made by illegal companies [4].

To improve the management at the control points of dredging construction sites, related to the entry to and exit from the construction sites, an automated monitoring system, vehicle license plate recognition (LPR), for dump trucks was developed. The system was built to provide permission for dump trucks’ entry and exit. This automated monitoring system can considerably reduce frontline personnel’s workload, increase construction efficiency, reduce the effect of human factors, and prevent errors.

This research process is based on the study goal, which was to facilitate an intelligent and highly efficient management system of dredging-related departments by providing a vehicle LPR system. In this study, the dredging operations’ status quo, situation, and current technology applications are identified through interviews with river management offices to understand the work and actual cases at control points in construction sites. The first part of building the truck LPR (TLPR) system involved collecting images of dump trucks and preprocessing the data. The second part consisted of selecting and analyzing the deep learning methods for recognizing vehicle license plates.

In the third part, three stages of the system process, localization, classification, and recognition, were built. To build the localization model, preprocessed data from previous steps were used as input. The localization model’s output was then used as an input in a convolutional neural network (CNN) structure to build a classification model. The classification model in this TLPR system would determine the number of characters on the license plates. The classification model’s outputs were then used to build a recognition model that was used to identify each character. Each model was assessed as to whether it reached the target performance.

The fourth part involved designing the process framework of an automated TLPR system for dump trucks. Finally, the best model for each stage was integrated to construct a TLPR system for the dump trucks, and the accuracy and operating speed of the system were assessed. The performance of the proposed system was compared with previous studies. Analyses of the study, the conclusions, and suggestions for future studies were then provided.

This paper forms a contribution to the current body of knowledge as it presents an objective approach to the TLPR system. It integrates the TLPR system in dredging operations, which already has an existing manual system. The scientific originality and contributions of this study lie in the ability to also recognize license plates with image noise like bad lighting and bad weather; reduced effects of obscured license plates (e.g., by sand and gravel); increased speed and accuracy of license plate inspections; reduced workload of personnel at the control point; and reduced probability of human errors.

## 2. Literature Review

### 2.1. ArtificiaI Intelligence (AI) Technologies for Smart Construction Sites

On conventional construction sites, human beings carry out most of the construction work. Engineering personnel with substantial experience are, in particular, responsible for regularly checking and maintaining equipment. Human checks, however, are usually costly and time-consuming. These checks also expose personnel to a complicated and unsafe working environment and affect public safety [5]. Various reasons for the low frequencies of equipment checks and maintenance leave much room for improvement in construction quality management methods [6].

Construction automation consists of combining computers, computation devices, and on-site robot technologies to simplify engineering tasks [7]. The resulting information also serves as a decision-making reference for all stakeholders in construction management. In equipment maintenance, for example, a data-driven system is developed for decision-making on equipment maintenance. The system implements quantitative decision-making models and integrates Building Information Modeling (BIM) and Geographic Information Systems (GIS) to support the data acquisition and update [8].

In the field of civil planning and management, the automatic recognition and classification of vehicle license plates using the CNN model and the detection of traffic flow with sensors enables the immediate, intelligent analysis of vehicles and road usage, as well as the modification of the intervals of traffic lights [9]. Hence, traffic engineers can understand the traffic conditions and arrange schedules for road maintenance and construction.

In this study, a smart dredging construction site is preliminarily designed, automated monitoring is performed using image recognition in critical areas, and further design and planning are conducted concerning construction site management.

### 2.2. Automated Monitoring Systems to Construction Management

In recent years, the omnipresence of surveillance cameras in cities has motivated the development of automated monitoring systems [10]. Automated monitoring systems, using captured images, are expected to reduce the workload of operators and facilitate the analysis of a considerable amount of data [11]. The analytical results of significant amounts of image data are used in early warning systems for buildings [12], identifying the activity of workforces [13] and machines, and assessing productivity [14].

An early warning system has four elements: knowledge of risk, monitoring and early warning, communication and dissemination, and response [15]. To prevent the deformation of roads and collapses in underground coal mining, Xu et al. constructed an automated monitoring and early warning system. The system captures roads’ tendency to deform, the deformation, and the damage to surrounding rocks, to warn about the instability of the latter [16].

In civil engineering, workforce performance management significantly influences construction progress and budget control [17]. Zhu et al. used images that were captured at construction sites in combination with feature extraction techniques to track workers and equipment, helping managers immediately to determine their positions and statuses [18].

Machinery and equipment also crucially affect construction productivity. The effective management and analysis of information about operations and related data help managers to optimize their operations and improve work efficiency [14,19]. In construction sites, surveillance videos and sensors have been used to identify activities and analyze excavators’ productivity during construction. Their motions have been automatically detected and tracked using machine learning or deep learning technologies [20,21,22]. Such systems can simplify the cumbersome process of human inspection and supervision, improve the collection of construction data, and help analyze construction practices [23].

This literature section presented automated systems’ benefits and technologies that are used in the field of construction.

### 2.3. Deep Learning for Vehicle License Plate Recognition

Vehicle LPR is widely employed in transportation [24,25,26] and comprises three steps: vehicle license plate localization, character segmentation, and character recognition [27,28,29]. However, several factors that are related to the images of license plates directly influence the difficulty of object detection and character recognition [30,31]. The images of vehicle license plates used in most studies are clear and non-oblique, which means the license plates directly face the camera [32,33], facilitating annotation and model training. The data augmentation method has also been used to increase the training sets’ diversity [34,35] and improve the model’s accuracy to recognize license plates.

Significant progress has been made by applying deep learning techniques to object detection. Among them, intense sampling of different areas of an image uniformly makes bounding boxes with various aspect ratios and dimensions. The bounding box results are then used with feature extraction using a CNN [36,37,38] for classification and regression. Typical vehicle LPR involve the You Only Look Once (YOLO) series [39,40,41,42,43,44] and single shot detector [45]. In most studies, YOLO models have exhibited high accuracy and recall, both above 98% [31,40,46,47]. They have also shortened inspections. Accordingly, YOLO network structures are frequently used in research as the ultimate models for object detection.

A wide range of methods of character segmentation exists. Recognition has been performed by classifying the segmented characters and inputting them into a CNN model for training [48,49], but the character segmentation is susceptible to image-related factors that cause difficulties [50,51]. The direct recognition of characters without segmentation has been developed [52,53]. Inspired by earlier work [54,55], Nguyen and Nguyen revised the CNN models to construct a network with three convolutional layers, three pooling layers, and two fully connected layers to solve the previous problem. They achieved a seven-digit vehicle LPR [56].

Captcha recognition techniques have successfully identified characters on the license plate [56]. JasonLiTW [57] aimed to solve captcha for booking tickets for the Taiwan High-Speed Rail (Simple Railway Captcha Solver (SRCS)). The author achieved a single character recognition rate as high as 99.39% and an overall successful recognition rate of 91.57% using data from 3000 images with data augmentation. Another well-known image recognition framework is Visual Geometry Group 16 (VGG16), which demonstrated an effective modeling network structure with little data [58,59,60]. Such networks commonly serve as a framework for models with less data, yielding highly accurate results.

The features of the images of the license plates on dump trucks in dredging operations that were obtained in this study, such as oblique plates, plates not directly facing the camera, various types of truck plates, short distances between the characters, and few data available, caused difficulty in detection, segmentation, classification, and model building. To overcome the challenges with minimal cost and disturbance on the current construction sites, the system must be conducted in three distinct stages: license plate localization, classification of the number of characters, and character recognition.

Specifically, modeling was carried out using the previously mentioned network structures (YOLO, CNN, SRCS, and VGG16) in each stage to train, validate, and test. This process was followed by estimating the accuracy and operating speed of the models. The performance was evaluated to identify the best model in each stage, which was ultimately integrated into a vehicle LPR system for dump trucks without replacing the current monitoring hardware on sites.

## 3. Research Methods

This section introduces the CNN and explains the diverse CNN structures and their comparative advantages. Lastly, the model validation methods and assessment criteria are elaborated.

### 3.1. Deep Learning Algorithms

#### 3.1.1. Convolutional Neural Network

A CNN is a feedforward neural network that consists of multiple convolutional layers, pooling layers, and fully connected layers. Through model training, the network’s parameters (weights and biases) are finely tuned, and the loss rate is evaluated to increase the accuracy of the model.

(1) Convolutional layer: In the convolutional layer, images from the previous layer are transformed into feature maps by applying filters. The height and width of the spatial axis of the generated feature map are the lengths that are obtained by the extraction of image information, while the depth is determined by the color (grayscale: 1, color: 3) format.

**Figure 1:**
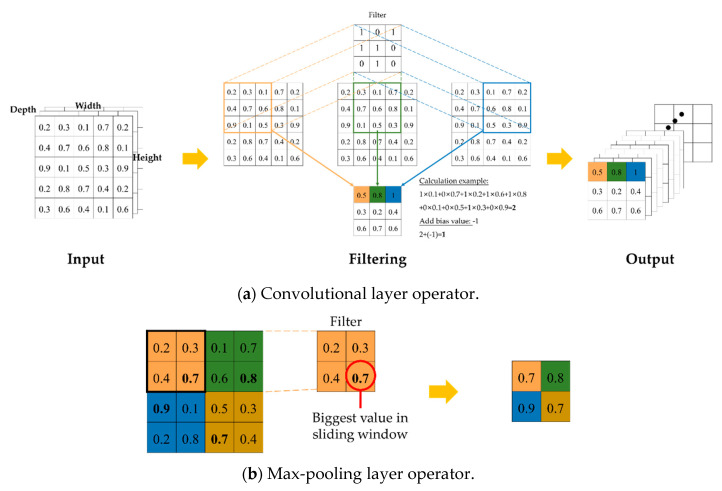
Convolutional layer and max-pooling layer operation.

A filter is also called a “kernel.” Each filter performs a computation on each small image unit. When the filter slides over the units, a corresponding computation is carried out to obtain the weights of the filter and images that overlap the filter. The total number is then calculated, and a bias is added, yielding a new feature map that is transmitted to the next layer, as shown in Figure 1a.

(2) Pooling layer: The pooling layer is used to remove unnecessary information from the feature map and preserve the most crucial information. The most popular sampling method is max-pooling. Figure 1b displays the corresponding calculation.

(3) Fully connected layer: This layer transmits the numerical values of the one-dimensional arrays that are the output from the flattening layer to the neurons. Classification is performed by observing the numerical output values of all or particular neurons.

(4) Dropout: This method can solve the problem of overfitting; it involves randomly dropping certain features that are output by the layer in the training period, reducing the complex co-adaptation among the neurons to improve the performance of the neural network; the dropout rate is generally set between 0.2 and 0.5, and at 0.5 in most cases.

(5) Activation and loss function: The activation function prevents the transmission of numerical values in the neural network only through a linear combination, which would reduce the neural networks’ ability to process complex features. The loss function is used to measure the neural networks’ performance against the training data, based on discrepancies between the model’s predictions and the actual values. The different problems correspond to different activation functions and loss functions, as provided in Table 1.

#### 3.1.2. You Only Look Once

YOLO is an object detection algorithm for neural networks; its advantages include rapidity and high accuracy. The neural network used in this study was the third version of YOLO, abbreviated as YOLOv3.

Before an image formally enters the neural network, YOLOv3 divides it into N × N grid cells. By the K-means method, nine types of anchor boxes are generated in each grid cell. The nine types of anchor boxes are divided into groups; each group contains three boxes, which change according to the size of the resulting feature map size before it zooms out to be the corresponding dimensions. The final product is a selected area that is considered to be the bounding box.

Darknet-53 (Table 2) is the backbone network structure of YOLOv3. The alteration involves removing the pooling layer and the fully connected layer of Darknet-53. Residual networks solve gradient problems, and feature pyramid networks (FPNs) [61] increase the ability of YOLOv3 to detect small objects, making it more stable.

#### 3.1.3. CNN-L3

CNN-L3 (Table 3) is a network structure that was generated by Nguyen and Nguyen [56]. Only 1000 vehicle license plate images were trained in this model, which can be considered a “low data situation”. With data augmentation, character recognition was achieved with high accuracy. Accordingly, the CNN-L3 was used in this study and fine-tuned for model training.

#### 3.1.4. Network Structure for Simple Solver of Railway Captcha for the Taiwan Railways Administration

JasonLiTW [57] established CNN to identify the captcha of the Taiwan Railways Administration. As shown in Table 4, it collected only 3000 captcha images, but data augmentation enabled both the classification model and the character recognition model to achieve high accuracy. The single character recognition rates and overall successful recognition rates of both models exceeded 90%.

#### 3.1.5. Visual Geometry Group 16 (VGG16)

VGG16 (Table 5) is one of the classic CNN models [62]. Its overall network structure is simple. However, since the three fully connected layers have numerous parameters, the resources that are required for computation are enormous, and such a deep network structure is not suitable for training with few data points. Accordingly, in this investigation, only the last fully connected layer was retained in a revised VGG16 model.

### 3.2. Criteria for Validating the Model and Assessing Errors

#### 3.2.1. Model Validation

**Figure 2:**
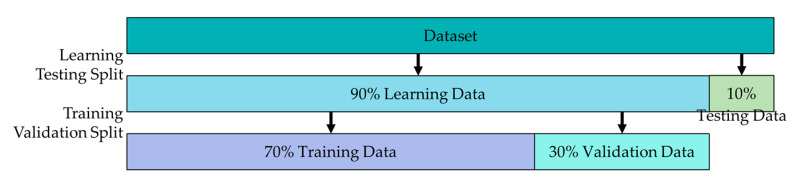
Holdout validation.

For model validation, this study used holdout validation to avoid overfitting during the model training. First, 90% of the raw data were used as the learning data, while the other 10% were used as the test data. The P% of the learning data was randomly used as the training data, for which (1−P)% of the data were used as the validation data. During model training, the validation data were repeatedly used to evaluate the model’s precision. The model’s parameters were continuously and slightly adjusted, ultimately increasing the model’s efficiency while mitigating the problem of overfitting. Figure 2 displays the adopted data segmentation for this study.

#### 3.2.2. Criteria for Assessing Accuracy

The following subsections present the three assessment stages of truck license plates: the accuracy of the truck license plate localization, the accuracy of the classification of the number of characters, and the accuracy of the character recognition.

(1) Assessment of truck license plate localization accuracy: In the object detection of the YOLO model series, the model assessment focuses on the numerical value of the mean average precision (mAP). The computation, definitions, and explanations of the mAP-related numerical values are presented below.

(a) Intersection over union: IoU is a metric of the accuracy of detection of the target object in an image. It is calculated by the ratio of the overlap area of the predicted box and the ground-truth box that is generated by the calculation model to the union area of the two boxes, as given by Equation (1).

(b) Precision: Precision is the ratio of the number of correctly predicted positive samples (true positives) to that of the samples predicted as positive (the sum of the numbers of true positives and false positives) by the model, as given by Equation (2). Table 6 presents the related references.

(c) Recall: Recall denotes the ratio of the number of correctly predicted positive samples (true positives) to the total number of positive samples (the sum of the numbers of true positives and false negatives), as demonstrated in Equation (3). Table 6 presents the related references.

(d) Average precision: A graph of the obtained numerical values of precision and recall is called a precision–recall curve (PR curve). The average precision (AP) is obtained by calculating the area under the curve. Finally, the average value of all classified AP results is the mAP. A higher mAP corresponds to greater precision of the model in framing the target object in each classification.

(1) $$\mathrm{IoU}=\frac{Detection\;Result\cap Ground\;Truth}{Detection\;Result\cup Ground\;Truth}$$

(2) $$\mathrm{Precision}=\frac{TP}{TP+FP}$$

(3) $$\mathrm{Recall}=\frac{TP}{TP+FN}$$

(2) Assessment of the accuracy of classifying the number of characters: The images of the truck license plates were input into the classification model to determine the number of characters. The license plates were classified into three categories with six, seven, and eight characters according to the current truck license plate type and coding rule. A vehicle license plate is deemed correctly classified when the number of characters in the image matches the predicted number. Equation (4) provides the accuracy of classification of the license plates by the number of characters.

(4) $$\mathrm{The}\;\mathrm{classification}\;\mathrm{accuracy}\;\mathrm{of}\;\mathrm{the}\;\mathrm{number}\;\mathrm{of}\;\mathrm{characters}=\frac{\mathrm{The}\;\mathrm{total}\;\mathrm{of}\;\mathrm{correctly}\;\mathrm{classified}\;\mathrm{truck}\;\mathrm{license}\;\mathrm{plates}}{\mathrm{The}\;\mathrm{total}\;\mathrm{of}\;\mathrm{truck}\;\mathrm{license}\;\mathrm{plates}\;\mathrm{being}\;\mathrm{classified}}\times 100\%$$

(3) Assessment of character recognition accuracy: This subsection presents two examples of predictions to facilitate the presentation of the overall successful recognition rate and single character recognition rate. Table 7 presents examples of the real images and the prediction of the trucks’ license plates.

(a) Overall successful recognition rate: The overall successful recognition rate is the percentage of individual truck license plates whose character locations and characters were predicted correctly to the total number of truck license plates for which the predictions were made, as given by Equation (5). A recognition was not deemed entirely correct if the characters on the plate had one or more errors in the determined character locations or the characters themselves. Regarding the examples in Table 7, the computed overall successful recognition rate was $\frac{1}{2}\times 100\%=50\%$.

(5) $$\mathrm{Overall}\;\mathrm{successful}\;\mathrm{recognition}\;\mathrm{rate}=\frac{\begin{array}{c} \mathrm{Number}\;\mathrm{of}\;\mathrm{individual}\;\mathrm{truck}\;\mathrm{license}\;\mathrm{plates}\;\mathrm{whose}\;\mathrm{character}\;\mathrm{locations}\;\mathrm{and}\\ \mathrm{characters}\;\mathrm{were}\;\mathrm{all}\;\mathrm{predicted}\;\mathrm{correctly} \end{array}}{\mathrm{Total}\;\mathrm{of}\;\mathrm{number}\;\mathrm{of}\;\mathrm{truck}\;\mathrm{license}\;\mathrm{plates}}\times 100\%$$

(b) Single character recognition rate: In some cases, the overall successful recognition rate did not precisely capture the predictive accuracy of the characters and their locations on the license plates, leading to inaccurate appraisal and comparison of the models. Equation (6) was used to solve this problem by assessing the single characters’ accuracy on the truck license plates. A prediction was deemed correct when the location and identity of the characters on a license plate were all predicted accurately. A prediction was deemed incorrect when either the localization of the characters or their identities were incorrectly determined. With respect to the examples in Table 7, the computed single character recognition rate was $\frac{6*2-2}{6*2}\times 100\%=83.33\%$ in all instances.

(6) $$\mathrm{Single}\;\mathrm{character}\;\mathrm{recognition}\;\mathrm{rate}=\frac{\begin{array}{c} \mathrm{Number}\;\mathrm{of}\;\mathrm{vehicle}\;\mathrm{license}\;\mathrm{plates}\;\mathrm{whose}\;\mathrm{character}\;\mathrm{locations}\;\mathrm{and}\\ \mathrm{characters}\;\mathrm{were}\;\mathrm{all}\;\mathrm{predicted}\;\mathrm{correctly} \end{array}}{\mathrm{Total}\;\mathrm{of}\;\mathrm{the}\;\mathrm{characters}\;\mathrm{to}\;\mathrm{be}\;\mathrm{predicted}}\times 100\%$$

## 4. Data Collection, Model Building, and System Development

**Figure 3:**
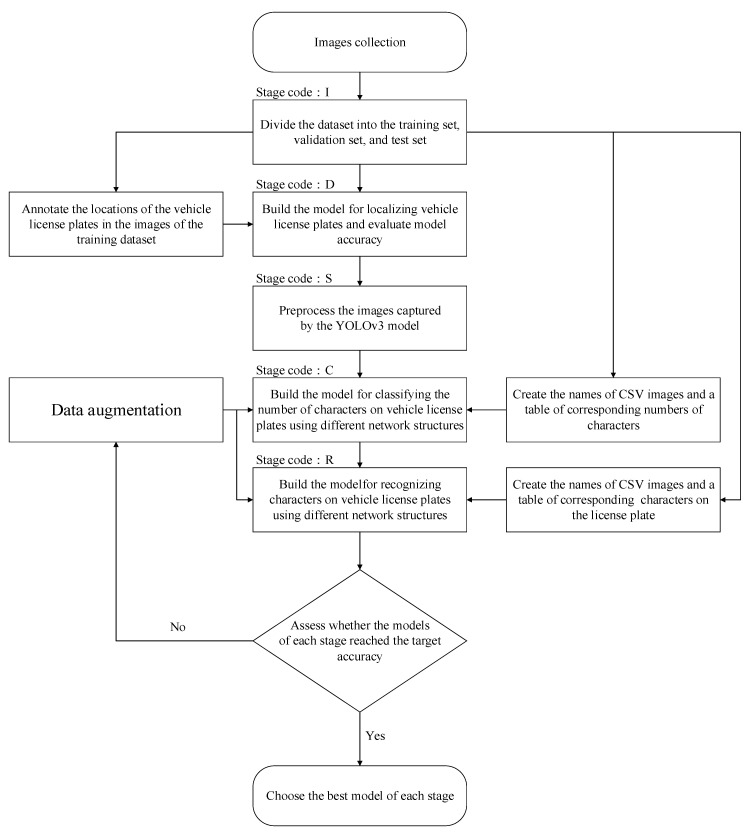
Model construction process at each stage of the truck license plate recognition (TLPR) system.

This section presents the software and hardware that were used in this study, the data collection and preprocessing of the TLPR system model, the construction, appraisal, and comparison of the models for each stage, the building and testing of the models, and the analysis and discussion of the results that are generated by the system. Figure 3 presents the procedures for building the models for each stage of the developed system.

### 4.1. River Dredging Management System

#### 4.1.1. Imaging of the License Plates of Dump Trucks

Images of the dump trucks in the Heping River Dredging Operation were obtained from the First River Management Office. They were generally of high quality with good camera angles and were captured in diverse weather and lighting. These images provided the training data for the TLPR models. A total of 5419 images and their truck license plate data were used for model training, validation, and testing.

#### 4.1.2. Image Data Preprocessing

A total of 5419 images were preprocessed differently in each stage. Table 8 presents the names and codes of the stages of the building of the TLPR system; it also presents the corresponding image data preprocesses and the numbers of images processed.

### 4.2. Individual Model Building and Validation

#### 4.2.1. Hardware and Software Specifications

Deep learning models were constructed using Python in Windows using Keras as the application programming interface (API) with TensorFlow, an open-source software library. Table 9 provides detailed specifications of hardware and software that were used herein.

#### 4.2.2. Data Usage and Model Construction

**Figure 4:**
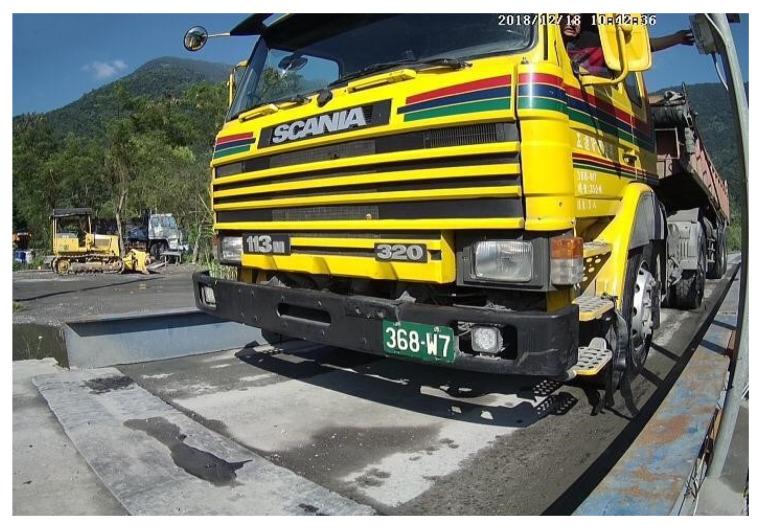
Truck entering a control point in daytime. Note: 1. The license plate number of the truck is pixelated because it is within the scope of protection of the law. 2. The image size is divided into two types: 708 × 480 pixels and 352 × 240 pixels.

A total of 5419 images of dump trucks were used in the models to locate the vehicle license plates, to determine the number of characters on the license plates, and to identify those characters. Noticeably, the images captured on-site were not originally for license plate recognition, thus increasing the complexity of the learning process. The images were then divided in a ratio of 9: 1 into the learning data and test data. The learning data included 4877 images in total, with 3414 images for training, 1463 images for validation, and 542 images for testing. Table 10 presents the quantities of image data used. Figure 4 shows an example of the dump truck image.

Table 11 presents the names of the stages in the system and the appraised and compared models. The model with the highest accuracy in each stage was selected as the optimal model. In this investigation, the standard accuracy was set to 95%; a model whose validation reached 95% in each stage was selected as the optimal model for that stage. Models whose accuracy did not meet that standard were retrained using data augmentation of the training data. Table 12 presents the setting for data augmentation.

**Figure 5:**
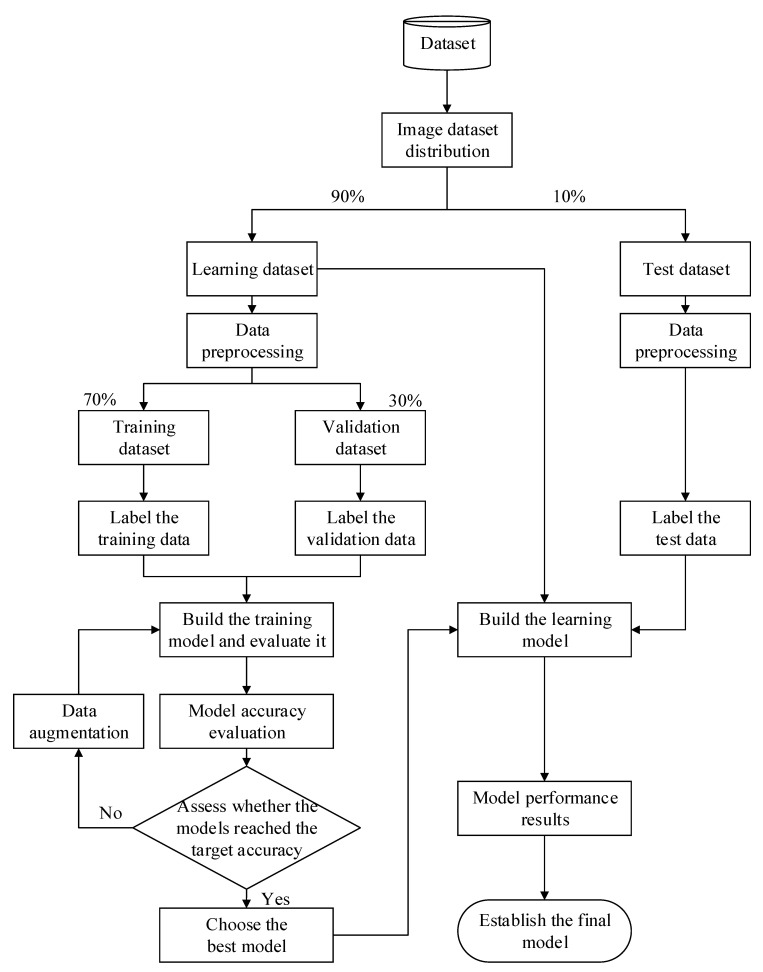
Flow chart of the construction of model at each stage.

Once the model with the highest accuracy was identified, the training and validation datasets were integrated as a whole dataset for the learning model. The network structure of the optimal model was used to train the learning model. Then a test was performed using the test dataset to evaluate the accuracy of the learning model. Figure 5 presents the model building, validation, and testing of the individual stages in detail.

##### Localizing Truck License Plates

**Figure 6:**
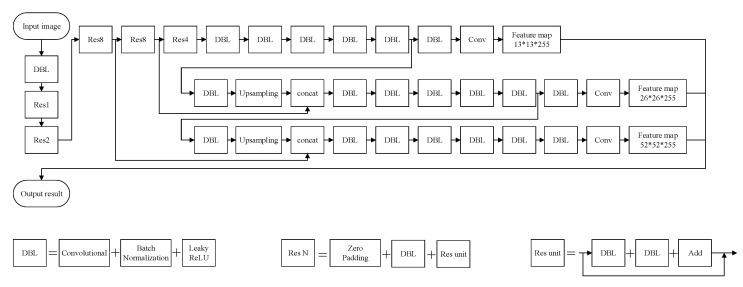
YOLOv3 network structure.

After a series of comparisons of the CNN models, the truck license plate localization model that was used in this study was YOLOv3, whose structure is shown in Figure 6. The results revealed that the mAP of YOLOv3 was 96.76%, and the recognition speed was as high as 0.025 s/image; the details are provided in Table 13. The assessed value met the set accuracy standard (95%). Therefore, both the training data and the validation data were used to train the learning model. During model testing, the mAP was as high as 97.14%, and the speed was 0.03 s/image; the details are presented in Table 13.

##### Classifying Truck License Plates by Number of Characters

In the stage of classification of the license plates by the number of characters, the CNN models of three structures, namely, C-CNN-L3, C-SRCS, and C-VGG16, were appraised and compared. Table 14 provides details of the three network structures. Their last output layers were identical, comprising a fully connected layer with the softmax activation function to perform multiclass, single-label classification, providing the categories that were related to the number of characters on the truck license plates—six, seven, or eight.

Table 15 provides the results of the model training. Before data augmentation, all three models had an accuracy of 79%, which did not meet the set target (95%). Accordingly, data augmentation was carried out during training. The C-CNN-L3 and C-SRCS models’ accuracies were consequently increased to 99.70% and 98.68%, respectively, while the C-VGG16 model’s accuracy only reached 81.08%. Hence, C-CNN-L3 was used as the optimal model of this stage in building the learning model.

Both the training data and the validation data were used to train the learning model; model training was performed in combination with data augmentation. As indicated by the test results in Table 15, the accuracy was 99.90% and the classification speed was 0.0315 s.

To examine the sensitivity of the model’s accuracy to the data augmentation parameters, the C-CNN-L3 network was used as the test structure. First, all parameters were set to the values in Table 12, and the model was trained. The accuracy was 99.80%. In the experiments, one of the parameters was removed, and only six parameter settings were used in model training. Table 16 provides the results. The ticked items in the table are the parameters used in the current model training. The goal of the test is to understand the effect of these parameters on the accuracy of the model.

The eight experiments demonstrated that, when the rescale parameters are removed, the accuracy is significantly reduced by approximately 18% to only 81.28%. The accuracy of the other experimental results exceeds 99%, so the rescale parameter can be inferred to have a much stronger impact on the accuracy of the character number classification model than the other six parameters, whereas the brightness, zoom, shear, height shift, width shift, and rotation parameters have similar impacts on the model. However, in the comparison of the six parameters, removing the zoom parameter still increases the model’s accuracy to 99.90%, optimizing model performance. Therefore, the zoom parameter is not used in training the learning model in its best state.

##### Recognizing Characters on Truck License Plates

In the license plate character recognition stage, the CNN models with three structures, R-CNN-L3, R-SRCS, and R-VGG16, were used for appraisal and comparison. Table 17 presents the network structures. Their last output layers were identical. Character prediction was carried out using the fully connected layers in combination with the softmax activation function, and a fully connected layer was constructed for the number of characters to be predicted.

The 35 characters were divided into three categories. The first category comprised the ten numbers from 0 to 9; the second category consisted of 24 English letters from A to Z (O and I are not used on license plates in Taiwan to avoid confusion because of their similarity to 0 and 1; the third category, “others”, consisted of one character, “-”.

Before data augmentation, the mean accuracy of the character recognition of all three models was only 30%. Data augmentation significantly increased the accuracy, with the R-CNN-L3 model performing the best, yielding a mean accuracy of at least 96% in recognizing six-, seven-, and eight-digit characters plates. Table 18 presents the details.

The R-CNN-L3 network structure was selected as the character recognition model for six, seven, and eight characters. Table 19 presents the testing results; the single character recognition rates of these models all exceeded 95%, the overall successful recognition rates ranged from 93% to 94%, and the recognition speeds per image ranged from 0.062 to 0.078 s. The single character recognition rate of the trained model of this stage reached the target.

### 4.3. Truck License Plate Recognition System

#### 4.3.1. System Integration

**Figure 7:**
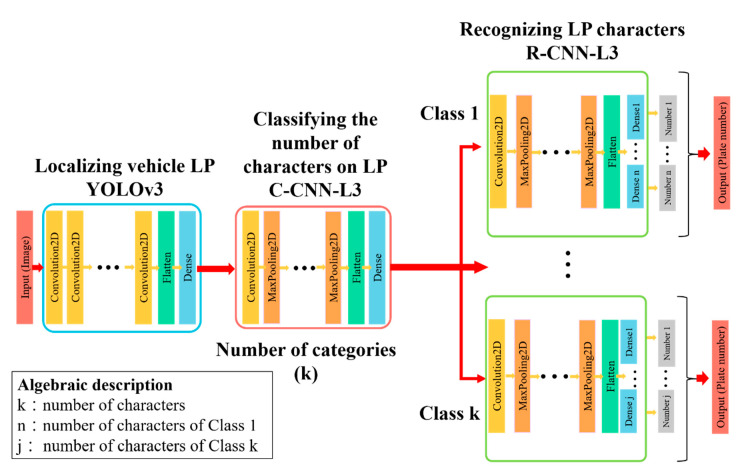
Network architecture of the TLPR system.

The TLPR system developed in this study was divided into three stages: license plate localization, classification by the number of characters, and character recognition. The optimal models of the three stages for testing were YOLOv3, C-CNN-L3, and R-CNN-L3. This section details how the optimal models were integrated into a TLPR system. Figure 7 shows the integrated network structure that was used in the real-time truck image recognition at the current dredging construction site.

Following the integration of the models of all stages, the overall TLPR system was evaluated. The assessed items were the overall successful recognition rate, the single character recognition rate, and the operating speed. Table 20 presents the results of the overall assessment of the system. They exhibited similarities with those of the individual stages in the previous section, revealing that the system integration in this study passed the test.

#### 4.3.2. System Analysis and Discussion

Based on the results of the model training and validation of the object detection, the image classification, and the digit recognition stages, network structures with fewer layers were more suitable for training the model using the data in this study. The accuracy of the models significantly increased following data augmentation because random changes in the brightness, shift range, and zoom of the training data allowed for the diversification of the training data. This method helped the model learn from data with more dimensions, increasing the accuracy and generalizability during testing.

The success of the procedures and framework for building the TLPR system was demonstrated by the results of the system testing. The overall success rate of the TLPR by the system was as high as 93.73%, the single character recognition rate was 97.59%, and the speed was 0.3271 s/image. Comparing the results of this research with those of prior studies on LPR, research on general LPR in various countries is discussed (see Table 21 for details). Despite dust adhering to truck license plates, equipment installation, the environment, and the amount of image data, the overall recognition was still 93.73%. Although the overall recognition rate needs to be improved, no prior study has recognized this complexity regarding truck license plates. This result thus reveals an improvement in engineering efficiency, the fair evaluation of license plates by the LPR system, and the reduction of risk of errors.

## 5. Use of the Dump Truck LPR System at Smart Dredging Construction Sites

The first subsection begins by presenting the planning of a smart dredging construction site and the design of an automatic control point. Then, the procedures for building a practical TLPR system is demonstrated in the second subsection. The third subsection provides a case study of dredging operations using the TLPR system. The final subsection analyzes the contributions of the TLPR system to automation in construction.

### 5.1. Smart Dredging Construction Site and Automation of Control Points

#### 5.1.1. Smart Dredging Construction Site Planning

**Figure 8:**
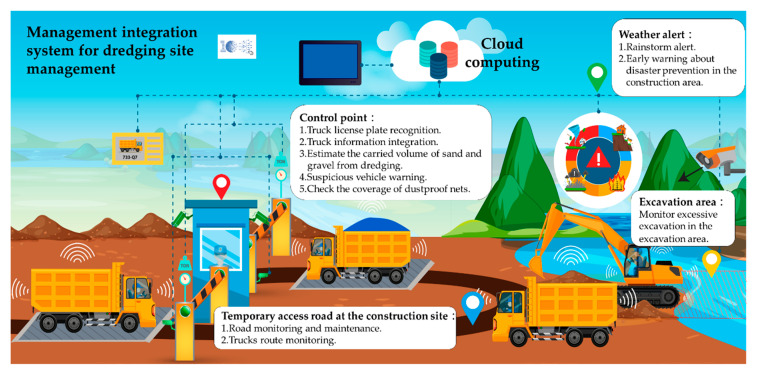
Smart dredging construction site planning.

The problems that are often encountered at dredging operation sites can be categorized. Relevant concepts around construction site management, equipment maintenance, and disaster prevention are referred to in the literature review. The concepts were used to plan a smart dredging construction site, as displayed in Figure 8. The figure presents the difficulties and problems encountered in the dredging construction area along with the potential benefits of smart construction site planning.

At the control point, the qualification inspections and information checks on hundreds of dump trucks are conducted manually every day. Therefore, mistakes in TLPR or threats and intimidation by unscrupulous operators can occur as personnel are under pressure to perform their duties quickly or as they are suffering from visual fatigue. Consequently, the tasks may not be performed fairly and efficiently, causing errors in dump truck entry and exit management.

According to the plan in this study, the license plates of the dump trucks were recognized using surveillance cameras at the control point, from which information is automatically associated with the corresponding vehicle data in the developed cloud system. The system used cameras to estimate the dredged sand and gravel volume, to check the dustproof nets, and to eliminate blind spots. Furthermore, the TLPR system can read images from the cameras around the construction area to identify suspicious vehicles and to inform managers when to implement related inspection measures.

Dump trucks travel on temporary access roads, but the long roads and lack of monitoring equipment make their status assessments difficult. The routing of vehicles is also a critical part of management; if the real-time location of a vehicle in a construction area cannot be determined precisely, then sand and gravel may get stolen, and construction accidents may occur without anyone’s knowledge.

The detection of cracks in roads by robots with automatic detection functions based on visual image recognition can reduce damage and extend the period of temporary road use. Regarding vehicle itineraries, a Global Positioning System (GPS) is used for tracking the current locations of vehicles that are immediately sent to the control station. In cases of unruly behavior on site, the patrolman is immediately instructed to check the site, reducing the opportunities for crime and accidents.

The interviews in this work revealed that although some river management offices have installed cameras to monitor the operation of the excavator, fair and accurate judgments of excessive excavation could seldom be made using real-time images based on the experience of dredge operators. Therefore, dredging disputes between dredging officials and construction bidders are relatively common, and these are unfavorable for construction.

Real-time surveillance images and related mathematical calculations were used to determine the angle and depth of the excavator’s motion and whether excessive excavation had occurred. In the case of excessive excavation, the system can remind dredge operators and help them make judgments rapidly, adopt related warning measures, or collect evidence and make decisions. Computer systems reduce conflicts concerning the excessive excavation of sand and gravel by supporting fair judgment.

While heavy rains followed by river swells and rapid flows commonly damage dredging areas, earthquakes followed by soil flow and landslides commonly affect the construction sites’ facilities. The upstream situation of the dredged river influences the dredging works downstream. Therefore, obtaining real-time information is effective in preventing these disasters in construction areas.

The automatic access to data from the Central Weather Bureau, regular dispatch of unmanned aerial vehicles (UAVs) upstream of a construction area to collect images, and automated early warning system for disaster prevention can all help personnel determine the probability of a negative impact by heavy rainfall in a construction area. All of the access, tools, and systems can provide immediate suggestions and warnings to prevent a disaster.

#### 5.1.2. Automated Design of the Control Point

**Figure 9:**
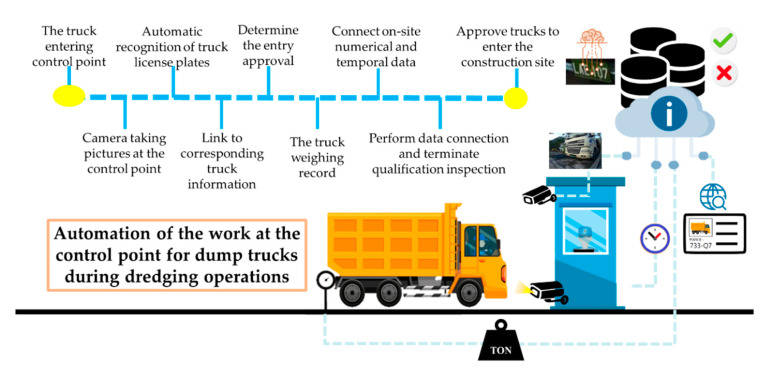
Design of the automation of the control point.

Figure 9 shows the preliminary plan of the automation procedure at a control point in the dredging operation. An automated vehicle LPR system for dump trucks that uses images from surveillance cameras at the entrance and the exit of the control point in the dredging area was developed; it also uses AI technologies and data on the dump trucks from the cloud. The system accesses on-site information to help personnel at the control point rapidly and precisely perform their tasks.

### 5.2. Recognition by the TLPR System

**Figure 10:**
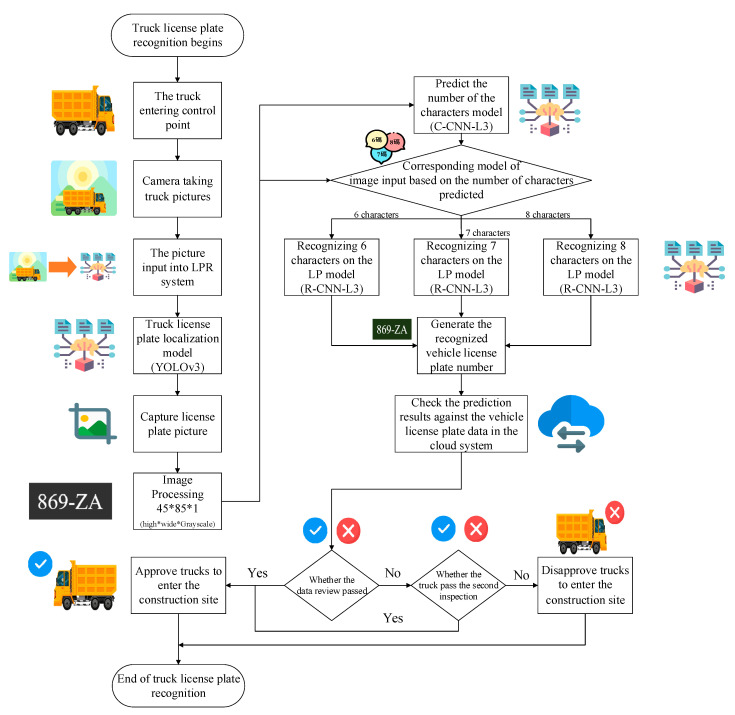
Flow chart of the license plate recognition system.

The TLPR system, based on the results in Section 4, can be used to control the entering and exiting of dump trucks to and from the control point at the construction site of a dredging operation. This system helps personnel at the control point recognize license plates and determine whether the trucks may enter and exit the construction site. Figure 10 displays the operating procedure of the system when the dump trucks enter the control point.

### 5.3. Case Study of Dredging Operations

#### 5.3.1. Effect of Weather and Lighting Factors on Recognition

**Figure 11:**
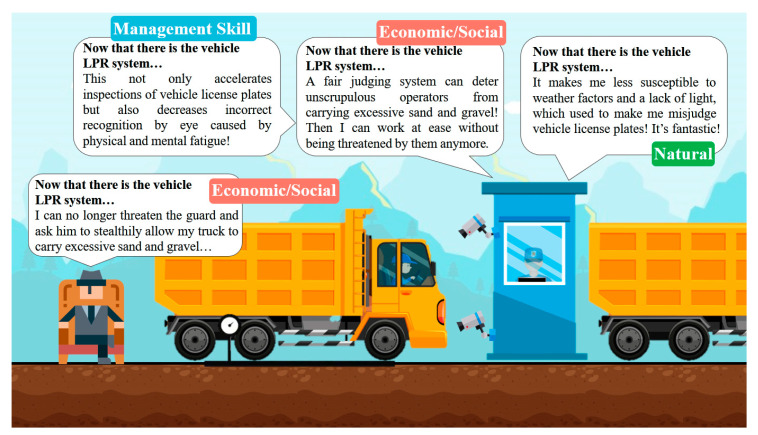
Case study of dredging operations.

The light in a construction site during dredging operations generally varies greatly. Use of the TLPR system that was developed in this study can effectively facilitate LPR for the vehicles of dredge operators in insufficient light and bad weather, reducing the extent to which environmental factors reduce the accuracy of recognition, as shown in Figure 11.

#### 5.3.2. Reducing Human Factors on Truck Entry and Exit

Because of the considerable benefits of selling dredged sand and gravel, unscrupulous operators often force personnel using threats, intimidation, violence, or bribes to allow their dump trucks to enter through the dredging operation’s control point gate. They steal sand and gravel, which they then transport away. Installing the TLPR system in this work can prevent unscrupulous operators from committing these criminal acts and reduce opportunities for them to do so, reducing the probability of threats to the personnel associated with dredging. The system enables police officers to rapidly collect evidence, making the dredging operations more transparent and reducing the probability of human errors, as shown in Figure 11.

#### 5.3.3. Alleviating Visual Fatigue Caused by Inspecting Numerous Vehicles

The frequency of trucks that transport sand and gravel entering during dredging operations is affected by the speed and quantity of the removal of sand and gravel by the excavators. By its accurate recognition of vehicle license plates, the system in this study can effectively reduce errors that are caused by the visual fatigue of personnel who inspect a very large number of vehicles. The system is also effective in situations like long queues by doing the check rapidly and smoothly. Figure 11 presents the details.

### 5.4. Contributions of the Proposed TLPR System to River Dredging Construction

#### 5.4.1. Security Guards at Control Points at Construction Sites

Incidents of sand and gravel theft during dredging operations occur continually. Security guards at control points often encounter violent intimidation and threats, and they are tempted with money from unscrupulous operators. These unscrupulous operators usually demand that their dump trucks be permitted to enter and exit the construction site without trouble so that they can steal sand and gravel. Use of the TLPR system at the control point to manage the permissions of dump trucks to enter and exit supports fair inspection of permissions, reduces the control of trucks entering and exiting by humans, helps to protect the security guards, and prevents unsafe working conditions.

Environmental factors, such as weather and light, often cause difficulties in vehicle LPR. The TLPR system herein can help security guards recognize license plate numbers, reducing human errors caused by environmental factors. On typical working days, the security guards at the control point must check and review the permissions associated with the license plates of hundreds of dump trucks. Long queues of dump trucks form if the checking rate is less than the rate of entering and exiting. The developed TLPR system is fast and accurate. It can compensate for the guards’ visual fatigue after long hours of work. Accordingly, the work stress at the control point can be largely minimized, and the effect of human limitations can be reduced.

#### 5.4.2. Dredging Authorities

Dredging officials may be subject to violent intimidation and threats or the temptation of money from unscrupulous operators who steal sand and gravel. Although dredging officials are not responsible for controlling the frontline management of dump trucks that enter and exit construction areas, they can affect the permissions of the dump trucks. The TLPR system herein can reduce the probability of control of truck entry and exit by human factors by supporting the fair and equitable inspection of permissions. It can improve safety in the environment where dredging officials work.

The priorities of construction managers in construction control are the time, cost, and efficiency of construction. Using the proposed TLPR system to manage the entry and exit of dump trucks at the control point during dredging operations can reduce the manpower needed and accelerate permission inspections, increasing the efficiency of the transportation of sand and gravel in construction. This system provides considerable benefits in construction execution and, for managers, it can decrease the risks associated with construction management.

#### 5.4.3. Departments of Police and Government Ethics

Since the human inspection of permissions of dump trucks to enter and exit the construction area depends on the inspector’s judgment at the control point, its results are susceptible to the influence of unscrupulous operators. Personnel may obey such people and allow their dump trucks to transport loads of sand and gravel without entry permission. The impartial judgment that is supported by the TLPR system herein reduces the corrupt human influence and helps to prevent the illegal conduct of unscrupulous operators. The system would help reduce the opportunities for stealing sand and gravel as well as the rate of crime.

When sand and gravel are stolen, police must collect evidence in the dredged area and conduct an overnight investigation. However, the police must allocate manpower, which is often unavailable. The TLPR system can record attempts by dump trucks to enter the construction site without permission. If the security guard does not prevent such trucks from entering or exiting, then the system can quickly provide reliable evidence for the police and government ethics departments, without the need for the police to allocate significant manpower.

## 6. Summary, Conclusions, and Suggestions

In dredging operations, control points at the entrances and exits are crucial for construction to progress smoothly. To reduce the possibility of a crime and incident from happening, personnel should accurately control and inspect dump trucks that enter and exit the construction area. To learn about the current execution of dredging operations and the difficulties encountered in the dredging operations’ process, interviews were carried out with staff at ten river management offices around Taiwan. Recent cases were analyzed, and a literature review was conducted.

The blueprint of a smart dredging construction site was planned following the summarization and organization of the information. With the automation of work at the control point as the goal, a vehicle LPR system for dump trucks (TLPR) was developed to reduce the inconvenience of performing tasks manually and the risks caused by human, natural, and management factors. The summaries of this study are as follows.

(1) In this study, the blueprint of a smart dredging construction site was provided. The blueprint was created in light of different tasks that are performed in the construction area. Image recognition technology and robot technology related to real-time information were used to improve the efficiency of construction execution while reducing the required labor force and the probability of error.

(2) An automated system was designed to support a dredging operation’s control point work. The goal was to make the work at the control point automatic, simpler, and more transparent. The focus of this study was on building and testing the TLPR system in a dredging operation.

(3) The TLPR system was constructed in four stages: collecting the image data, designing the system process and structure, building and testing models of the individual stages, and finally integrating the optimal models of the individual stages based on the system structure to produce a single system. The system was then used in dredging operations and the relevant situations were simulated. The TLPR process was divided into three main stages: license plate localization, classification of the number of characters, and character identification.

(4) During model training, the YOLOv3 model was used for truck license plate localization, with an mAP of 96.76% and a speed of 0.025 s/image. The classification by the number of characters was compared with three network structures. C-CNN-L3, which was trained with data augmentation, was the optimal model of this stage, with an accuracy of 99.70%. Character recognition was similarly appraised and compared using three network structures. For the R-CNN-L3 model with data augmentation, the single character recognition rates with six, seven, and eight characters on a license plate were all above 95%. Thus, the optimal network structure for each stage, YOLOv3, C-CNN-L3, and R-CNN-L3, was used to train the learning model.

(5) When the learning model was being tested individually for each stage, the mAP of YOLOv3, the model used to locate truck license plates, was 97.14, and the speed of detection was 0.03 s/image. The accuracy of C-CNN-L3, the model for classifying plate images by the number of characters, was 99.90%, and the speed was 0.0315 s/image. The overall successful recognition rates of R-CNN-L3, the character recognition model, for plates with six, seven, and eight characters all exceeded 93.16%; the single character recognition rate exceeded 97.80%; and the recognition speed was between 0.0624 and 0.0781 s/image. The learning models of the individual stages were then integrated into a TLPR system. Testing revealed that the system’s overall successful recognition rate and single character recognition rate were 93.73% and 97.59%, respectively, and the detection speed was 0.3271 s/image.

(6) Based on the model’s performance, network structures with fewer layers shows better performance, implying the fewer layer networks were more suitable for the data in this study. The performance of the investigated models was also influenced by data augmentation, especially the rescale parameter, which shows better accuracy with sensitive tests. This study also found that, in the system construction process, image quality critically influences the development and accuracy of the TLPR system. Improving the functions, resolution, installation layout, and lighting of the on-site hardware in the construction area can accelerate recognition and make it more accurate, supporting dump truck management.

The TLPR system in this work can help personnel to manage the entry and exit of dump trucks during dredging operations by facilitating license plate recognition. The system would inspect dump trucks’ entry and exit permissions and prevent unscrupulous operators from coming into the construction area and stealing sand and gravel, all while aiding police departments to collect evidence rapidly when required. The automated system would also prevent inspection activities from being affected by factors related to weather and light conditions, thereby reducing errors. It also reduced the possibility of personnel misjudgments, providing a range of benefits to security guards at the control point, dredging officials, and police departments.

This paper presented an objective approach for the TLPR system in dredging operations in the contribution of the LPR system. Dredging operation, which originally has an existing manual checking and inspection system, was integrated into the building of the developed TLPR system with the intention of making a smart construction site. The unique contributions of this study lie in the ability of the developed system to recognize noise-covered license plates (e.g., by sand, grave, bad weather, or bad lighting); increase the computation speed and accuracy of the license plate inspections; reduce the probability of human errors; and assist security guards at the control point.

In the future, multiple sets of TLPR systems that use different algorithms with high accuracy can be used jointly to read the same vehicle license plate, reducing the probability of error recognition. To increase the positioning speed and accuracy of the localizing vehicle license plates model, future studies should use a relatively novel target detection network like EfficientNet, YOLOv4, or YOLOv5.

A function that warns of suspicious vehicles can be developed by installing vehicle LPR systems elsewhere in the construction area. When vehicles that are recognized as unqualified enter said area, the patrolman is immediately notified for pursuit and capture. Functions of the vehicle LPR system that support dredging operation tasks can be further developed, improving dredging operations toward the goal of a smart construction site.

## Figures and Tables

**Table 1:** Problem type and corresponding activation function and loss function.

Problem Type	Activation Function	Loss Function
Binary classification	Sigmoid	Binary_crossentropy
Multiclass, single-label classification	Softmax	Categorical_crossentropy
Multiclass, multilabel classification	Sigmoid	Binary_crossentropy
Regression to arbitrary values	Linear	Meansquared error
Regression to values between 0 and 1	Sigmoid	Meansquared error or binary_crossentropy

**Table 2:** Darknet-53 network structure.

Type	Filters	Size	Output
Convolutional	32	3 × 3	256 × 256
Convolutional	64	3 × 3/2	128 × 128
Convolutional	32	1 × 1	
Convolutional	64	3 × 3	
Residual			128 × 128
Convolutional	128	3 × 3/2	64 × 64
Convolutional	64	1 × 1	
Convolutional	128	3 × 3	
Residual			64 × 64
Convolutional	256	3 × 3/2	32 × 32
Convolutional	128	1 × 1	
Convolutional	256	3 × 3	
Residual			32 × 32
Convolutional	512	3 × 3/2	16 × 16
Convolutional	256	1 × 1	
Convolutional	512	3 × 3	
Residual			16 × 16
Convolutional	1024	3 × 3/2	8 × 8
Convolutional	512	1 × 1	
Convolutional	1024	3 × 3	
Residual			8 × 8
Avg Pool		Global	
Connected		1000	
Softmax			

**Table 3:** CNN-L3 network structure.

Layer	Type	Network
1	Input	128 × 64
2	Convolutional	48@5 × 5
3	Max-pooling	2 × 2
4	Convolutional	64@5 × 5
5	Max-pooling	1 × 2
6	Convolutional	128@5 × 5
7	Max-pooling	2 × 2
8	Fully Connected	2048
9	Fully Connected	36 × 7

**Table 4:** Simple Railway Captcha Solver (SRCS) network structure.

Layer	Type	Network
1	Input	200 × 60
2	Convolutional	32@3 × 3
3	Convolutional	32@3 × 3
4	Batch Normalization	–
5	Max-pooling	2 × 2
6	Dropout	0.5
7	Convolutional	64@3 × 3
8	Convolutional	64@3 × 3
9	Batch Normalization	–
10	Max-pooling	2 × 2
11	Dropout	0.5
12	Convolutional	128@3 × 3
13	Convolutional	128@3 × 3
14	Batch Normalization	–
15	Max-pooling	2 × 2
16	Dropout	0.5
17	Convolutional	256@3 × 3
18	Batch Normalization	–
19	Max-pooling	2 × 2
20	Flatten	–
21	Dropout	0.5
22	Fully Connected	34 × 5

**Table 5:** VGG16 network structure.

Layer	Type	Network
1	Input	200 × 60
2	Convolutional	64@3 × 3
3	Convolutional	64@3 × 3
4	Max-pooling	2 × 2
5	Convolutional	128@3 × 3
6	Convolutional	128@3 × 3
7	Max-pooling	2 × 2
8	Convolutional	256@3 × 3
9	Convolutional	256@3 × 3
10	Convolutional	256@3 × 3
11	Max-pooling	2 × 2
12	Convolutional	512@3 × 3
13	Convolutional	512@3 × 3
14	Convolutional	512@3 × 3
15	Max-pooling	2 × 2
16	Convolutional	512@3 × 3
17	Convolutional	512@3 × 3
18	Convolutional	512@3 × 3
19	Max-pooling	2 × 2
20	Flatten	–
21	Fully Connected	4096
22	Fully Connected	4096
23	Fully Connected	1000

**Table 6:** Confusion matrix.

Confusion Matrix		Actual	
		True	False
**Predicted**	**Positive**	True Positive (TP)	False Positive (FP)
	**Negative**	False Negative (FN)	True Negative (TN)

**Table 7:** Example of the evaluation of character recognition system.

	Items	Actual LP Photo	Actual LP No.	Predicted LP Image	Predicted LP No.
Prediction No.					
Prediction 1		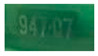	947-Q7	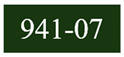	941-07
Prediction 2		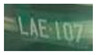	LAE-107	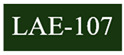	LAE-107
Prediction 3		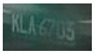	KLA-6703	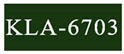	KLA-6703

**Table 8:** Pre-processing image data for each stage.

Stage	Stage Code	Image Data Pre-Processing	Number of Preprocessed Images
Image dataset distribution	I	The 5419 images are divided into 90% learning data sets and 10% test data sets, and all image names are encoded.	Learning data: 4877 images Test data: 542 images
The stage of vehicle license plate localization	D	The 4877 images are divided into training and validation data according to the 70/30 rule. Use the LabelImg software to mark the plate location in every image.	Train data: 3414 images Validation data: 1463 images
Capture images and standardize their formats	S	Standardize the dimensions of the images of vehicle license plates captured from the YOLOv3 model to 45 × 85 pixels, and change their color into greyscale.	Learning data: 4877 images
The stage of classification of the number of characters	C	The 4877 images are divided into training and validation data according to the 70/30 rule.	Train data: 3414 images Validation data: 1463 images
The stage of character recognition	R	The data set is the same as the classification of the number of characters stage, without additional image data preprocessing.	Train data: 3414 images Validation data: 1463 images

Note: Because all the license plate images framed by YOLOv3 do not exceed 45 × 85 pixels, this size is used as the standard for the unified specification to avoid image distortion caused by excessive stretching of the image.

**Table 9:** Specifications of software and hardware.

Software and Hardware Equipment	Specification
CPU	INTEL Core i7-8700 3.2 GHz CPU
Motherboard	ASUS
GPU	NVIDIA GeForce RTX2080Ti-11G GDDR6
RAM	32 GB DDR4-2666 RAM
Application	CUDA version 10.1.120
Platform	Windows 10
Programming	Python

**Table 10:** Image information in every dataset.

	No. of Characters	6 Characters	7 Characters	8 Characters	Total Images
Dataset					
Learning Data		3389 images	240 images	1248 images	4877 images
Training Data		2372 images	168 images	874 images	3414 images
Validation Data		1017 images	72 images	374 images	1463 images
Test Data		387 images	38 images	117 images	542 images

**Table 11:** Correspondence matrix between the system stage and evaluation models.

	Stage	The Stage of Vehicle License Plate Localization (D)	The Stage of Classification of the Number of Characters (C)	The Stage of Character Recognition (R)
Model				
YOLOv3		✓	-	-
C-CNN-L3		-	✓	-
C-SRCS		-	✓	-
C-VGG16		-	✓	-
R-CNN-L3		-	-	✓
R-SRCS		-	-	✓
R-VGG16		-	-	✓

**Table 12:** Data augmentation parameter setting.

Parameter	Command	Range
Brightness	brightness_range	0.3–1.3
Rotation	rotation_range	0–10
Shift	width_shift_range	0–0.1
	height_shift_range	0–0.1
Zoom	zoom_range	0–0.1
Shear	shear_range	0–0.1
Rescale	rescale	1/255

**Table 13:** Outputs of YOLOv3 model.

**Training Phase**				
**Model**	**Category**	**mAP**	**Loss Value**	**Speed** **(s/image)**
YOLOv3	1	96.76	2.87	0.025
**Test Phase**				
**Model**	**Category**	**mAP**	**Loss Value**	**Speed** **(s/image)**
YOLOv3	1	97.14	2.58	0.03

**Table 14:** Network architectures for classifying the truck license plates by the number of characters thereon.

	Network Architecture	C-CNN-L3	C-SRCS	C-VGG16
No. of Layers				
Input layer		Input (45 × 85 × 1)	Input (45 × 85 × 1)	Input (45 × 85 × 1)
1		Convolutional (48@5 × 5)	Convolutional (32@3 × 3)	Convolutional (32@3 × 3)
2		Max-pooling (2 × 2)	Convolutional (32@3 × 3)	Convolutional (32@3 × 3)
3		Convolutional (64@5 × 5)	Batch Normalization	Max-pooling (2 × 2)
4		Max-pooling (2 × 2)	Max-pooling (2 × 2)	Convolutional (64@3 × 3)
5		Convolutional (128@5 × 5)	Dropout (0.5)	Convolutional (64@3 × 3)
6		Max-pooling (2 × 2)	Convolutional (64@3 × 3)	Max-pooling (2 × 2)
7		Dropout (0.5)	Convolutional (64@3 × 3)	Convolutional (128@3 × 3)
8		Flatten	Batch Normalization	Convolutional (128@3 × 3)
9		2500	Max-pooling (2 × 2)	Convolutional (128@3 × 3)
10		–	Dropout (0.5)	Max-pooling (2 × 2)
11		–	Convolutional (128@3 × 3)	Dropout (0.5)
12		–	Convolutional (128@3 × 3)	Convolutional (512@3 × 3)
13		–	Batch Normalization	Convolutional (512@3 × 3)
14		–	Max-pooling (2 × 2)	Convolutional (512@3 × 3)
15		–	Dropout (0.5)	Max-pooling (2 × 2)
16		–	Convolutional (128@3 × 3)	Convolutional (512@3 × 3)
17		–	Batch Normalization	Convolutional (512@3 × 3)
18		–	Max-pooling (2 × 2)	Convolutional (512@3 × 3)
19		–	Flatten	Max-pooling (2 × 2)
20		–	Dropout (0.5)	Dropout (0.5)
21		–	–	Flatten
Output layer		Fully connected layer (3, Softmax)		

**Table 15:** Accuracy of the models in classifying the plate images by number of characters.

**Training Phase**			
Networks		Accuracy	
		Not using data augmentation (%)	Using data augmentation (%)
C-CNN-L3		79.56	99.70
C-SRCS		79.63	98.68
C-VGG16		79.56	81.08
**Test Phase**			
	Performance	Accuracy (%)	Speed (s/image)
Model			
C-CNN-L3		99.90	0.0315

**Table 16:** Effect of data augmentation on the classification accuracy.

	Parameter	Brightness	Zoom	Shear	Height Shift	Width Shift	Rescale	Rotation	Accuracy (%)
No									
1		✓	✓	✓	✓	✓	✓	✓	99.80
2			✓	✓	✓	✓	✓	✓	99.80
3		✓		✓	✓	✓	✓	✓	99.90
4		✓	✓		✓	✓	✓	✓	99.80
5		✓	✓	✓		✓	✓	✓	99.80
6		✓	✓	✓	✓		✓	✓	99.70
7		✓	✓	✓	✓	✓		✓	81.28
8		✓	✓	✓	✓	✓	✓		99.79

**Table 17:** Network architectures for recognizing the characters on truck license plates.

	Networks	R-CNN-L3	R-SRCS	R-VGG16
Number of Layers				
Input layer		Input (45 × 85 × 1)	Input (45 × 85 × 1)	Input (45 × 85 × 1)
1		Convolutional (48@5 × 5)	Convolutional (32@3 × 3)	Convolutional (32@3 × 3)
2		Max-pooling (2 × 2)	Convolutional (32@3 × 3)	Convolutional (32@3 × 3)
3		Convolutional (64@5 × 5)	Batch Normalization	Max-pooling (2 × 2)
4		Max-pooling (2 × 2)	Max-pooling (2 × 2)	Convolutional (64@3 × 3)
5		Convolutional (128@5 × 5)	Dropout (0.5)	Convolutional (64@3 × 3)
6		Max-pooling (2 × 2)	Convolutional (64@3 × 3)	Max-pooling (2 × 2)
7		Dropout (0.5)	Convolutional (64@3 × 3)	Convolutional (128@3 × 3)
8		Flatten	Batch Normalization	Convolutional (128@3 × 3)
9		–	Max-pooling (2 × 2)	Convolutional (128@3 × 3)
10		–	Dropout (0.5)	Max-pooling (2 × 2)
11		–	Convolutional (128@3 × 3)	Dropout (0.5)
12		–	Convolutional (128@3 × 3)	Convolutional (512@3 × 3)
13		–	Batch Normalization	Convolutional (512@3 × 3)
14		–	Max-pooling (2 × 2)	Convolutional (512@3 × 3)
15		–	Dropout (0.5)	Max-pooling (2 × 2)
16		–	Convolutional (128@3 × 3)	Convolutional (512@3 × 3)
17		–	Batch Normalization	Convolutional (512@3 × 3)
18		–	Max-pooling (2 × 2)	Convolutional (512@3 × 3)
19		–	Flatten	Max-pooling (2 × 2)
20		–	Dropout (0.5)	Dropout (0.5)
21		–	–	Flatten
Output layer		Fully connected layer (35, Softmax)		

		Fully connected layer (35, Softmax)		

Note: The fully connected layer of the output layer sets the number of fully connected layers according to the number of characters (6, 7, and 8 codes).

**Table 18:** Accuracy of the models in recognizing the characters on truck license plates.

	Character No.	Single Character Recognition Rate		
Networks		6 Characters	7 Characters	8 Characters
R-CNN-L3		96.34%	98.23%	96.97%
R-SRCS		77.80%	82.78%	79.65%
R-VGG16		35.37%	72.61%	89.74%

Note: Data augmentation—brightness; rotation; shift; zoom; shear; and rescale.

**Table 19:** Results of the test of the learning model in recognizing characters on truck license plates.

Character No.		6 Characters		7 Characters		8 Characters	
No. of test image		387 images		38 images		117 images	
	**Accuracy**	**Overall** **successful recognition rate**	**Single** **character** **recognition rate**	**Overall** **successful recognition rate**	**Single** **character** **recognition rate**	**Overall** **successful recognition rate**	**Single character recognition rate**
**Model**							
CNN-L3		94.06%	97.80%	94.74%	99.25%	93.16%	98.83%
Speed		0.0624 s		0.0673 s		0.0781 s	

**Table 20:** Overall evaluation of the TLPR system.

	Items	No. of Test Image	Overall Successful Recognition Rate (%)	Single Character Recognition Rate (%)	Speed (s/image)
System					
TLPR system		542 images	93.73	97.59	0.3271

**Table 21:** Comparison of results of the LPR system in various countries.

Article	Countries	Technique	Overall Successful Recognition Rate	Number of Characters
Segmentation-Free Vehicle License Plate Recognition Using CNN [53]	China	YOLOv2; RDNet	99.34%	7 characters (30-class characters)
A New Convolutional Architecture for Vietnamese Car Plate Recognition [56]	Vietnam	CNN-L3	97.84%	7 characters (30-class characters)
A Hybrid KNN-SVM Model for Iranian License Plate Recognition [63]	Iran	KNN-SVM	97.03%	8 characters (22-class characters)
Artificial neural networks based vehicle license plate recognition [64]	Turkey	Canny edge; ROI; ANN	95.36%	–
Deep Learning System for Automatic License Plate Detection and Recognition [48]	USA Taiwan	Canny edge; CNN	Caltech 94.8% AOLP 95.1%	– (37-class characters)
Automatic license plate recognition via sliding-window darknet-YOLO deep learning [40]	Taiwan	YOLO	78%	6 characters (36-class characters)
This study	Taiwan	YOLOv3; CNN-L3	93.79%	6–8 characters (35-class characters)

## Data Availability

The data are not publicly available due to privacy policy.

## Nomenclature

AI	Artificial Intelligence
AP	Average Precision
ANN	Artificial Neural Network [1]
API	Application Programming Interface
BIM	Building Information Modeling
CNN	Convolutional Neural etwork [2]
CNN-L3	Convolutional Neural Network with Three Feature Stages [3,4]
CPU	Central Processing Unit
C-CNN-L3	Convolutional Neural Network for Classification of the Number of Characters with Three Feature Stages
C-SRCS	Simple Railway Captcha Solver for Classification of the Number of Characters
C-VGG16	Visual Geometry Group 16 for Classification of the Number of Characters
FN	False Negative
FP	False Positive
FPN	Feature Pyramid Network [5]
GIS	Geographic Information System
GPU	Graphics Processing Unit
GPS	Global Positioning System
IoU	Intersection over Union
KNN-SVM	K-Nearest Neighbors and the Multi-Class Support Vector Machines [6]
LPR	License Plate Recognition
mAP	Mean Average Precision
PR Curve	Precision-Recall Curve
RAM	Random-Access Memory
RDNet	Combination of Dense Convolutional Network (DenseNet) and Residual Network (ResNet)’s Advantages [7]
R-CNN-L3	Convolutional Neural Network for Character Recognition with Three Feature Stages
R-SRCS	Simple Railway Captcha Solver for Character Recognition
R-VGG16	Visual Geometry Group 16 for Character Recognition
SRCS	Simple Railway Captcha Solver
TLPR	Truck License Plate Recognition
TN	True Negative
TP	True Positive
UAV	Unmanned Aerial Vehicle
VGG16	Visual Geometry Group 16 [8]
YOLO	You Only Look Once [9]
YOLOv2	You Only Look Once Version 2
YOLOv3	You Only Look Once Version 3
YOLOv4	You Only Look Once Version 4
YOLOv5	You Only Look Once Version 5
